# Vitamin D as a Potential Player in Immunologic Control over Multiple Myeloma Cells: Implications for Adjuvant Therapies

**DOI:** 10.3390/nu14091802

**Published:** 2022-04-26

**Authors:** Piotr Kulig, Karolina Łuczkowska, Anna Bielikowicz, Debora Zdrojewska, Bartłomiej Baumert, Bogusław Machaliński

**Affiliations:** Department of General Pathology, Pomeranian Medical University, 70-111 Szczecin, Poland; piotrkulig@interia.eu (P.K.); karolina.luczkowska@pum.edu.pl (K.Ł.); bielikowicz.anna@gmail.com (A.B.); zdrojewska.debora@gmail.com (D.Z.); bbaumert@pum.edu.pl (B.B.)

**Keywords:** multiple myeloma, vitamin D, vitamin K, immunomodulation, adjuvant therapy, peripheral neuropathy

## Abstract

Multiple myeloma (MM) is a plasma cell malignancy with multifactorial etiology. One of the underlying mechanisms is immune system dysregulation. Immunotherapy is being widely introduced into various MM treatment protocols. Nevertheless, little is known about boosting the immune system with supportive treatment. Although classical actions of vitamin D (VD) are very well established, their non-classical actions related to the modulation of the immune system in MM are still a subject of ongoing research. In this literature review, we intend to summarize research conducted on VD and MM, both in vitro and in vivo, with particular emphasis on immune system modulation, the induction of the differentiation of malignant MM cells, synergic activity with anti-MM drugs, and MM-associated peripheral neuropathy.

## 1. Introduction

Multiple myeloma (MM) is a non-common hematological cancer that accounts for approximately 10% of all blood malignancies [1], with an annual incidence rate of approximately 5/100,000 [2]. MM arises from plasma cells and typically develops from a premalignant state—monoclonal gammopathy of undetermined significance—through smoldering MM and eventually to symptomatic MM that requires immediate treatment [3]. The prognosis for MM patients is nowadays significantly better compared with the past as a result of the constant progress in pharmacotherapy and the more profound understanding of the underlying abnormalities on a molecular level. Moreover, therapy has become more personalized since the treatment regime is adjusted to a patient’s overall condition and their molecular and cytogenetic abnormalities. Nevertheless, the necessity for the constant improvement of currently existing chemotherapy regimens and supportive treatment is self-evident.

Immune system dysfunctions seem to play a crucial role in MM development and progression. Multiple mechanisms and therapeutic approaches were described in the literature [4]. The immunological processes responsible for resistance to the applied chemotherapeutic agents and the development of peripheral neuropathy in MM are also gradually becoming better understood [5,6,7]. Vitamins, with a particular emphasis on vitamin A and D, have a well-established impact on the immune system [8]. Vitamin D (VD) is thought to possess potentially beneficial actions in prevention and supportive treatment for a huge variety of conditions [9].

In this review, we discuss the immunomodulatory effects of vitamin D supplementation as an adjuvant therapy for MM and its potential broader application in improving patient outcomes. Although there is a shortage of research being carried out on VD in the management of MM, we intend to provide a thorough insight into the existing literature with a special emphasis on immune mechanisms and the modulation of cellular growth and differentiation.

## 2. Methods

We searched the PubMed, PubMed Central, Scopus, Web of Science, Embase, and Google Scholar databases in detail for papers associated with MM, and vitamins D and K and their analogs. We used the following keywords: multiple myeloma vitamin D; plasma cell dyscrasia vitamin D; multiple myeloma vitamin K; plasma cell dyscrasia vitamin K; monoclonal gammopathy vitamin D; monoclonal gammopathy vitamin D; multiple myeloma cholecalciferol; plasma cell dyscrasia cholecalciferol; monoclonal gammopathy cholecalciferol; multiple myeloma calcidiol; plasma cell dyscrasia calcidiol; monoclonal gammopathy calcidiol; multiple myeloma calcitriol; plasma cell dyscrasia calcitriol; monoclonal gammopathy calcitriol; multiple myeloma 25(OH)D3; plasma cell dyscrasia 25(OH)D3; monoclonal gammopathy 25(OH)D3; multiple myeloma 1,25(OH)2D3; plasma cell dyscrasia 1,25(OH)2D3; monoclonal gammopathy 1,25(OH)2D3; stem cell transplantation vitamin D; stem cell transplantation vitamin K; stem cell transplantation cholecalciferol; stem cell transplantation calcidiol; stem cell transplantation calcitriol, stem cell transplantation 25(OH)D3; and stem cell transplantation 1,25(OH)2D3. In our review, we included all in vitro studies, clinical trials, and other types of studies, such as retrospective cohort studies, which were exclusively focused on multiple myeloma, myeloma cell lines, and the abovementioned vitamins.

## 3. Vitamin D

### 3.1. General Overview

VD is not a vitamin per se, but acts as a steroid prohormone, which is synthesized in the skin by a process dependent on exposure to ultraviolet radiation. In addition, VD can be obtained from dietary sources [10]. In order to become physiologically active, VD undergoes two hydroxylation reactions. The first, in which 25(OH)D3 is synthesized, occurs in the liver. 25(OH)D3 (calcidiol) subsequently undergoes the second hydroxylation in the kidneys, predominantly in the proximal tubule. The final product of these reactions is 1,25(OH)2D3 (calcitriol), which is the most biologically active form of VD [11].

The classical role of VD is the regulation of calcium and phosphate homeostasis, and it is therefore crucial for bone metabolism and health. In the intestine, VD promotes calcium absorption, while in kidneys, 1,25(OH)2D3 regulates renal calcium reabsorption and phosphate loss. Biological actions of 1,25(OH)2D3 are mediated by intracellular vitamin D receptor (VDR) [12]. VD deficiency results in rickets in children and osteomalacia in adults [10]. Although the role of VD in calcium homeostasis is very well established, its biology is still the subject of ongoing research.

### 3.2. Non-Classical Actions

Although the classical actions of VD are very well characterized, so-called non-classical actions, i.e., not directly related to calcium and phosphate metabolism, are the subject of intensive ongoing research. VDR is expressed in the vast majority of human cells [13]. Therefore, one may presume that VD might modulate the functions of multiple cell types via regulating gene expression, exhibiting numerous pleiotropic effects. Besides genomic action mediated by VDR, VD also exhibits non-genomic effects, believed to be mediated by membrane receptors [14,15]. Non-genomic actions are predominantly associated with activating various signaling molecules, the subsequent generation of second messengers, and the opening of ion channels [16].

Serum concentration of 25(OH)D3 is the best indicator of VD status. It reflects total VD produced in the skin as well as that obtained through food and supplements. What is more, 25(OH)D3 has a long circulating half-life of 15 days [17]. Since many cells in the human body express VDR, VD is believed to modulate cell proliferation, differentiation, and apoptosis. On top of that, it also regulates gene expression associated with the modulation of cell growth, inflammation, and neuromuscular and immune functions [17]. VDR is expressed not only in healthy tissues but has also had its presence confirmed in malignant cells. For instance, according to Huss et al., a high expression of VDR in invasive breast tumors is associated with favorable prognostic factors and a low risk of breast cancer death. Thus, it was concluded that a high VDR expression is a positive prognostic factor [18].

Although the serum concentration of 25(OH)D3 is the best indicator of VD status, under particular circumstances, low 25(OH)D3 may not always reflect actual VD deficiency. In the acute-phase response, lower serum concentrations of 25(OH)D3 were observed [19,20,21]. This phenomenon should be taken into account when considering VD status in MM patients, since malignancy is associated with inflammatory response [22]. Nevertheless, whether a decrease in serum 25(OH)D3 level is a component of an acute-phase reaction remains a point of controversy, since there are studies that do not confirm such observations [23,24].

### 3.3. Vitamin D in Multiple Myeloma

A serum concentration of 25(OH)D3 below the reference interval is prevalent among MM patients. In order to measure 25(OH)D3 status in this subset of patients, Graklanov et al. conducted a study in which they enrolled 37 patients (19 women, 18 men) with a median age of 68 years. Serum 25(OH)D3 levels were assessed on the first day of hospitalization, prior to treatment initiation. All enrolled patients had serum 25(OH)D3 concentrations below the reference interval. One patient had VD insufficiency with serum 25(OH)D3 levels between 20 and 30 ng/mL, while the remaining 36 patients had a 25(OH)D3 concentration below 20 ng/mL (VD deficiency). 25(OH)D3 below 10 ng/mL (severe VD deficiency) was reported in 81% of patients [25]. The objective of another study was to investigate serum 25(OH)D3 levels in patients newly diagnosed with non-Hodgkin lymphoma/diffuse large B-cell lymphoma, multiple myeloma, and chronic lymphocytic leukemia. The obtained results demonstrate that a serum level of 25(OH)D3 below the reference range is a very common condition among patients with hematological malignancies, particularly in MM. All 103 patients had serum levels of 25(OH)D3 below 30 ng/mL. It should be emphasized that 81% of MM had 25(OH)D3 levels below 10 ng/mL (defined as severe VD deficiency), in comparison to 32.3% of non-Hodgkin lymphoma/diffuse large B-cell lymphoma patients and 28.1% of chronic lymphocytic leukemia patients [26]. In the study conducted by Maier and colleagues, the mean 25(OH)D3 level in patients with MM and bone lesions was 14.8 ng/mL (±6.3 ng/mL). The authors concluded that it is of utmost clinical importance to assess 25(OH)D3 levels in cancer patients, especially those with, or at high risk of, developing metastatic bone disease [27]. Badros et al. assessed 25(OH)D3 status in 100 MM patients. Among the enrolled individuals, 40% had VD deficiency, defined by serum 25(OH)D3 levels ≤ 36 nmol/L (14.42 ng/mL), 35% had VD insufficiency, defined by levels of 36–75 nmol/L (14.42–30.05 ng/mL), and only 25% had sufficient VD levels, defined as ≥75 nmol/L (30.05 ng/mL) [28]. The aforementioned studies indicate that a low serum level of 25(OH)D3, which may indicate a VD deficiency, is a very common condition among patients suffering from hematological malignancies, with a particular emphasis on MM. Moreover, polymorphisms were found in genes encoding VDR associated with an increased risk of MM. The same study concluded that several VDR gene polymorphisms may be considered as a molecular marker of the risk of MM [29]. Another study described a link between the FokI polymorphism in the *VDR* gene and the increased susceptibility to the development and progression of MM in the ethnic Kashmiri population [30].

#### 3.3.1. Vitamin D and Peripheral Neuropathy in Multiple Myeloma

Peripheral neuropathy (PN) is a common condition among MM patients and may occur as a treatment-related complication or as a result of the disease itself. Oortgiesen et al. investigated the relationship between VD deficiency and PN prevalence. The results suggest an association between low VD levels and the occurrence of PN, which, in turn, might imply a neuroprotective effect of VD [31]. In another study, serum levels of 25(OH)D3 were measured in 111 MM patients treated with either thalidomide or bortezomib. It was established that 25(OH)D3-deficient MM patients were more likely to have severe PN of both motor and sensory types. The obtained results show that the severity of PN appears to be bound to VD deficiency, which, in turn, provides justification for monitoring serum VD levels and eventual supplementation [32]. Similar results were obtained in the descriptive study conducted by Nath et al. Namely, 41 MM patients were enrolled to investigate VD status and its relationship with clinical outcomes. Patients with VD deficiency had a higher likelihood of PN compared with their non-VD-deficient counterparts [33]. It needs to be stressed that a correlation does not prove the causative association between low VD status and the occurrence and severity of PN in MM. In order to further explore this area, clinical trials should be conducted.

#### 3.3.2. In Vitro Studies

The assumption that VD may play an important role in MM adjuvant treatment has a well-established molecular background, especially when VD is administered in combination with novel drugs. It is worth noting that VD might activate macrophages and enhance their activity [34]. Eicher et al. conducted an in vitro study in which they investigated whether lenalidomide improves the anti-MM activity of myeloma-associated macrophages (MAMs) elicited by MOR202 (monoclonal antibody against CD38) and whether the vitamin D pathway is part of its modulatory effects. First, they established that MAMs have a reduced capacity to convert 25(OH)D3 into bioactive 1,25(OH)2D3. Subsequently, they found that lenalidomide restores VD metabolism in MAMs and therefore increases their ability to convert 25(OH)D3 into its biologically active form, 1,25(OH)2D3. Not only does 1,25(OH)2D3 enhance the tumoricidal activity of MAMs, but it also targets MM cells under its influence. After 48 h of exposure to 25(OH)D3, MM cells exhibited a higher expression of CD38. Since the efficacy of monoclonal antibodies is partly dependent on target antigen expression, it was subsequently investigated if there was a higher propensity to bind to the surface of MM cells after treatment with 1,25(OH)2D3. After labeling MOR202 with a fluorescent dye, enhanced binding of the antibody to the surface of MM cells after incubation with 25(OH)D3 was found. The study concluded that the active form of VD not only acts on the effector cells but also increases the vulnerability towards anti-CD38 antibodies by enhancing CD38 expression on MM cells [35].

On the other hand, in another in vitro study, it was demonstrated that MM cells may undergo trans-differentiation into osteoclast-like cells. VD may participate in the aforementioned lineage switching, thus enhancing the bone lytic capabilities of malignant plasma cells in MM. However, the authors are far from concluding that VD supplementation could be harmful in vivo [36].

Ozdemir and colleagues conducted a study that investigated the effects of dexamethasone, all-trans retinoic acid, 1,25(OH)2D3, and interferon alpha on FO mouse myeloma cells (non-immunoglobulin-secreting myeloma cell line). Cells were incubated with the aforementioned agents alone and in six drug combinations (dexamethasone + 1,25(OH)2D3, dexamethasone + all-trans retinoic acid, dexamethasone + interferon, 1,25(OH)2D3 + all-trans retinoic acid, 1,25(OH)2D3 + interferon, and interferon + all-trans retinoic acid) and were compared with the control. In conclusion, the authors stated all-trans retinoic acid, interferon, vitamin D3, and particularly dexamethasone as having significant effects on FO mouse myeloma cells, decreasing cell count and increasing apoptosis. Nevertheless, it should be noted that the decrease in cell count in the 1,25(OH)2D3 group was not statistically significant compared with the control [37].

Bortezomib (BTZ) implementation was a turning point in MM therapy. This agent acts as a proteasome inhibitor, a crucial molecule in regulating the intracellular protein degradation pathway [38]. It is well established that BTZ itself significantly improved the clinical outcomes of MM patients. Nevertheless, it was not elucidated if VD and BTZ combined together exhibited synergic effects. Kaiser et al. investigated the role of VD in the BTZ-induced stimulation of the osteoblast differentiation. They cocultured myeloma LP-1 and OPM-2 cell lines with primary human mesenchymal stem cells and human osteoblasts. Coculturing the primary human mesenchymal stem cells and human osteoblasts with MM cells resulted in an inhibition of the vitamin D-dependent differentiation of osteoblast precursors. Treatment with bortezomib led to a moderate increase in osteoblastic differentiation in primary human mesenchymal stem cells and human osteoblasts. This effect was substantially increased when 25(OH)D3 (5 nM) was added to the medium. Moreover, they demonstrated a stronger increase in VDR-mediated signaling in cells stimulated with 25(OH)D3 and BTZ in comparison to 25(OH)D3 alone. Based on this in vitro study, the authors hypothesized that maintaining adequate VD levels in MM patients could contribute to the osteoblastic differentiation of bone precursor cells. This may be of clinical relevance and provides the framework for clinical trials to investigate the combined effects of bortezomib and vitamin D on bone metabolism in MM [39].

Bisphosphonates are widely implemented in MM treatment in order to reduce the risk of skeletal complications [40]. In an ex vivo study, the interaction between VD and pamidronate was investigated. MM cells (line H929) were exposed to VD alone and VD combined with pamidronate. The researchers reported a marked synergistic interaction between pamidronate and 1,24(OH)2D2, resulting in enhanced antiproliferative effects in the tested MM cell line [41].

In addition to VD, which, under physiological circumstances, is synthesized in the skin or obtained from the diet, there are synthetic analogs that exhibit similar pharmacological actions yet with limited hypercalcemic potential. These may be, for instance, used for the treatment of secondary hyperparathyroidism. Kumagai et al. investigated whether 19-nor-1,25-dihydroxyvitamin D2 (paricalcitol) exhibits similar antitumor activity to VD. They cultured several cancer cell lineages, among them myeloma cell lines (RPMI-8226, ARH-77, and NCI-H929), with paricalcitol. The antitumor activity of paricalcitol was detected and this effect occurred predominantly via the induction of apoptosis [42].

EB1089 is a VD analog with well-established antitumor properties, and with reduced hypercalcemic effects in vitro and in vivo in comparison to l,25(OH)2D3. It has also been under investigation in MM. Puthier et al. conducted a study which concluded that EB1089 alone and in combination with dexamethasone is able to inhibit the growth of MM cells in vitro [43].

Park and colleagues studied the effect of EB1089 on MM cell lineages (ARH-77, NCI-H929, and RPMI8226). The results suggest that EB1089 can inhibit the proliferation of human myeloma cells, especially NCI-H929 cells, via a G(1) block in association with the induction of p27 and a reduction in CDK2 activity [44].

In another in vitro study, the human myeloma cell line NCI-H929 was cultured with EB1089. The effects of EB1089 on cell growth, the cell cycle, MAPKs (mitogen-activated protein kinase) activity, and apoptosis were investigated. EB1089 exhibited a dose-dependent inhibition of cell growth in this cell line. In summary, it was established that EB1089 inhibits the cell growth of NCI-H929 cells by not only inducing G1 arrest but also by triggering apoptosis by the activation of caspase 3 and p38 MAP kinase [45].

The same team investigated the effects of EB1089 and TGF-ßl on cell proliferation, cell cycle distribution, and apoptosis in various human myeloma cell lines: NCI-H929, ARH-77, RPMI8226, and U266. While TGF-ßl alone could not inhibit the proliferation of any of the tested myeloma cells, a synergistic effect between EB1089 and TGF-ßl was observed in NCI-H929 cells. Thorough analysis revealed that the effect occurred through the apoptotic pathway [46].

The cited in vitro studies revealed promising results. Research conducted on cell lineages is important, as it provides a molecular basis for the design of future studies and enables the understanding of specific mechanisms and interactions in a stable environment. On the other hand, their conclusions need to be verified in clinical trials as laboratory conditions do not necessarily reflect the complexity of interactions in the human organism.

#### 3.3.3. Clinical Trials

It is presumed that VD status may play a role in MM prognosis and morbidity. One study involved 148 newly diagnosed MM patients whose serum 25(OH)D3 levels were assessed within 14 days of diagnosis. The aim of the study was to examine the relationship between VD deficiency and the presentation of MM at diagnosis. Individuals with VD deficiency had higher serum CRP, serum creatinine, and International Staging System stage at the time of diagnosis. Interestingly, there were no differences in skeletal morbidity, defined as lytic lesions, long bone fractures, and vertebral compression fractures, detected between subjects with VD deficiency or sufficiency. Although the authors stated that vitamin D deficiency may be a predictor of poorer outcome in MM, they also hypothesize an alternative explanation. For instance, subjects with more advanced disease at diagnosis may have a reduced caloric intake, with a consequent reduction in dietary vitamin D intake [47].

High-dose chemotherapy (HDCT) followed by autologous stem cell transplantation (ASCT) is a preferred consolidation treatment option for MM patients who fulfill prespecified criteria [48]. Eicher and colleagues investigated the role of VD status in terms of overall survival (OS) and progression-free survival (PFS) in MM and lymphoma patients who underwent ASCT preceded by HDCT. The cut-off point between low and normal 25(OH)D3 status was set at 52 nmol/L. The researchers established that overall mortality was significantly lower (*p* = 0.0191) in the normal 25(OH)D3 group. Simultaneously, the PFS was longer in MM patients with normal 25(OH)D3 status compared with the low 25(OH)D3 group (*p* = 0.0412). Although OS did not differ significantly between normal and low 25(OH)D3 patients with lymphomas (*p* = 0.1242), significant differences were documented in the MM patients in favor of the normal 25(OH)D3 group (*p* = 0.049). Moreover, VD status was proven to be an independent prognostic variable for OS. Although the results of this study suggest a beneficial influence of normal 25(OH)D3 concentrations, attention should be paid to the observational nature of the study. Thus, it could not be determined whether VD supplementation would improve patient outcomes [49].

In another study, the relationship between VD status and OS was investigated in cohorts of white and African American MM patients. In this research, the threshold for VD deficiency was established at below 20 ng/mL (50 nmol/L). For white patients, OS was significantly lower in patients with VD deficiency compared with those with normal VD levels. In addition, on multivariate analysis, VD deficiency status turned out to be a strong independent predictor of OS after adjustment for age and stage at diagnosis. On the other hand, this association was not confirmed in the African American subgroup. Nevertheless, the study shows the importance of screening for VD deficiency at the time of MM diagnosis. Furthermore, it highlights the different impact of VD status across race, where white patients with the deficiency had much worse outcomes [50].

In a randomized clinical control trial, Raoufinejad et al. investigated the clinical outcomes of patients with MM, Hodgkin lymphoma, and non-Hodgkin lymphoma. A total of 80 patients were enrolled (22 MM patients in calcitriol group and 22 in the placebo group) and randomly allocated to either the calcitriol or placebo group. Oral capsules of calcitriol 0.25 ug or placebo were administered three times daily from day 0 to 30. The obtained results show that the recovery rate of the absolute lymphocyte count was significantly higher in the calcitriol group than the placebo. In addition, the two-year relapse-free survival was significantly higher in the calcitriol than the placebo group (*p* = 0.03). A significant limitation of this study is the low number of enrolled MM patients [51].

Laroche et al. investigated the impact of 25(OH)D3 status on bone metabolism in MM patients after ASCT. They enrolled 39 patients to their study. Several parameters, including VD status, were measured at the time of ASCT and 12 months later. Patients in the vitamin D-deficient group had higher serum PTH levels than those in the vitamin D-sufficient group. Nevertheless, biochemical bone markers were identical in both groups at the time of ASCT and one year later. The study concluded that VD deficiency does not impair the biochemical markers of bone metabolism in patients with MM, before and after ASCT. However, it is important to note that the enrolled group was relatively small, and the cut-off point for VD deficiency was established at 20 ng/mL. In addition, mean 25(OH)D3 levels were relatively low, i.e., 23 ± 4 ng/mL and 25.7 ± 4 ng/mL, before and after ASCT, respectively. Similarly, a limitation of this study is the small number of patients enrolled and its observational nature [52].

In a retrospective cohort study, 83 MM patients were enrolled, including 17 patients with newly diagnosed MM. Subjects were unselected, and therefore, represented a broad spectrum of time points of MM diagnosis and treatment. The effect of VD supplementation using laboratory parameters was also examined. Lower 25(OH)D3 levels (<10 ng/mL) were associated with a higher number of plasma cells in the bone marrow. Vitamin D supplementation has been shown to increase the number of erythrocytes (3.8 to 4.0 × 10^6^/μL, *p* = 0.004), hemoglobin (11.8 to 12.3 g/dL, *p* = 0.039), and leukocytes (4.9 to 5.8 × 10^3^/μL *p* = 0.011), whereas the number of thrombocytes (200.5 to 175.2 × 10^3^/μL, *p* = 0.036) decreased. The study concluded that the supplementation of VD appears to be safe in MM patients. However, dosing strategies should be adjusted for particular MM patients. Whether normalizing 25(OH)D3 levels in these patients actually improved clinical outcomes requires assessment in clinical trials, in particular, because a limitation of the study was that the cohort represented a broad spectrum of time points of MM diagnosis and treatment. Therefore, the aforementioned improvements could be an effect of the applied therapies [53].

### 3.4. Non-Classical Effects of Vitamins K and D and Their Potential Common Role in Multiple Myeloma

There is a common belief that VD should be supplemented in parallel with vitamin K (VK). Historically, it was thought that the biological function of VK is exclusively bound to hemostasis. Over time, due to extensive research, knowledge of the physiology of VK has expanded. Currently, it is proven that VK, similarly to VD, exhibits multiple pleiotropic effects [54,55]. In particular, VK2 is being thoroughly investigated, since this isoform contributes to the vast majority of processes under the control of extrahepatic vitamin K-dependent proteins (VKDPs) [56]. In the Rotterdam Study, which was a prospective population-based study, it was established that adequate menaquinone (VK2) dietary intake is associated with a protective effect against coronary heart disease, which could be mediated by the inhibition of arterial calcification [57]. Another important facet connected to VK2 and extrahepatic VKDPs is bone metabolism and health. Shiraki et al. enrolled 241 osteoporotic patients into a 24-month randomized open-label study. The control group (without treatment; *n* = 121) and the vitamin K2-treated group (*n* = 120), which received 45 mg/day vitamin K2 (menatetrenone) orally, were followed for lumbar bone mineral density and the occurrence of new clinical fractures. The study concluded that VK2 treatment in osteoporosis successfully inhibited the occurrence of new bone fractures and maintained lumbar bone mineral density. Although the underlying background for the abovementioned results has not been entirely elucidated, it has been hypothesized that osteocalcin may play an important role [58].

It should be noted that VK2 prevents the calcification of tissues. It is particularly beneficial with regard to cardiovascular health. Matrix Gla protein is expressed by vascular smooth muscle cells and is a potent calcification inhibitor. Post-translational modification of Matrix Gla protein is a VK-dependent process which is crucial in the prevention of calcification [59]. Furthermore, one might hypothesize that this particular effect would presumably decrease the potential of VD to trigger hypercalcemia when supplemented in higher doses.

In in vitro studies, VK, particularly the VK2 isoform, exhibited anticancer activity. For instance, it was demonstrated that VK2 triggers bladder cancer cells’ death via autophagy [60]. Similar effects were depicted in hematological malignancies. Yokoyama et al. showed that VK2 exerts cellular death via both autophagy and apoptosis in leukemic cells. However, it was more prominent when the cells were protected from rapid apoptotic death by a high expression level of Bcl-2 [61]. In another study, it was shown that VK2 can induce apoptosis in various leukemic cell lineages. In addition, VK2 enhanced the effect of all-trans retinoic acid. Surprisingly, it was also found that VK1 did not have any effect on leukemic cells [62]. Xu and colleagues investigated whether VK1, VK2, and VK3 have cytotoxic effects against T lymphoblastoid leukemia cells. The results show that all tested isoforms exhibited cytotoxic effects on the tested cell lineages [63].

VK was also investigated in MM. However, there is a lack of clinical trials, and research is limited to studies on myeloma cell lines. VK2 in vitro diminishes the proliferation of MM cells. According to Otsuki and colleagues, who cultured ten different MM cell lineages with VK2, this effect occurs predominantly via the induction of apoptosis [64]. Similar to the VD analogs, VK analogs also demonstrate anticancer effects. Plumbagin is a VK3 analog. In an in vitro study, U266 MM cells were cultured with plumbagin, BTZ, and thalidomide in different combinations. The study showed that the agent has the ability to suppress STAT3 activation, inhibit IL-6-induced STAT3 and JAK1 phosphorylation, downregulate the expression of cyclin D1, Bcl-xL, and VEGF, inhibit cell proliferation, and induce apoptosis. Moreover, it enhances the effect of bortezomib and thalidomide. Of note is also the lack of toxicity of plumbagin [65].

VD and VK may exhibit synergic effects, especially in terms of bone [66,67] and cardiovascular health [68,69]. Therefore, it may be hypothesized that those vitamins may enhance their biological effects when administered together in MM. Therefore, it appears reasonable to supplement VD and VK in MM in order to boost immune mechanisms and enhance the efficacy of chemotherapy. However, further in-depth studies are necessary to address this issue.

## 4. Conclusions

Emerging evidence, based on both in vitro studies and clinical trials, suggests that VD supplementation may exert beneficial effects as an adjuvant treatment for MM patients, particularly in combination with novel chemotherapeutic agents such as lenalidomide, bortezomib, and anti-CD38 antibodies.

There is engaging evidence, predominantly based on in vitro studies, that VD may be of clinical relevance in MM management as an adjuvant therapy. Moreover, the non-classical actions of VK and its synergy with VD in relation to bone and cardiovascular health should also be taken into consideration. Thereby, it could be hypothesized that the administration of both vitamins could enhance their effect in MM. Nevertheless, there is a shortage of prospective clinical trials. Therefore, we herein propose to conduct prospective clinical trials focusing on the supplementation VD and VK, including their analogs, in MM patients. We also suggest exploring the use of higher doses of vitamins than in the general population, especially in combination with novel MM drugs. It should be of particular interest to study the effect of the combination of VD and VK2 MK-7 in MM both in vitro and in vivo, since the combination of the abovementioned vitamins may exert a synergistic effect.

## Data Availability

Not applicable.
